# *Toxoplasma gondii* molecular detection and phylogenetic analysis in hemodialysis patients from Khuzestan, Southwest Iran

**DOI:** 10.1186/s41182-024-00585-5

**Published:** 2024-02-13

**Authors:** Saba Yarahmadi, Jasem Saki, Reza Arjmand, Heshmatolah Shahbazian

**Affiliations:** 1 Department of Medical Parasitology, School of Medicine, Ahvaz Jundishapur University of Medical Sciences, P. O. Box 6135743337, Ahvaz, 393-61155 Iran; 2 Health Research Institute, Infectious and Tropical Diseases Research Center, Ahvaz Jundishapur University of Medical Sciences, P.O. Box 6135743337, Ahvaz, 393-61155 Iran; 3 Department of Nephrology, Golestan General Hospital, Diabetes and CKD Research Center, Ahvaz Jundishapur University of Medical Sciences, P.O. Box 6135743337, Ahvaz, 393-61155 Iran

**Keywords:** Hemodialysis, Nested-PCR, Sequence, *Toxoplasma gondii*

## Abstract

**Background:**

The diagnosis and genetic characterization of *Toxoplasma gondii *(*T. gondii*) infection can make a significant influence to the prevention of the dangerous consequences of toxoplasmosis, particularly in immunocompromised people.

**Objective:**

The aim of this investigation was to assess the frequency and genotyping of *T. gondii* in blood samples of patients with hemodialysis.

**Materials and methods:**

In the current investigation, a total of 379 blood samples were taken from subjects with hemodialysis who were referred to teaching hospital of Ahvaz in the southwest of Iran. The samples were evaluated using the Nested PCR by targeting the B1 gene, and then, sequencing and phylogenetic tree were constructed.

**Results:**

*T. gondii* DNA was found in 112 (29.55%) of the blood samples by Nested PCR. Amplicons from *T. gondii* revealed high identity with GenBank sequences. The phylogenetic analysis revealed that all sequences were closely related to Type I of *T*. *gondii*.

**Conclusion:**

Because of the high incidence of toxoplasmosis with type I prevalent in hemodialysis patients, we recommend a systematic screening for toxoplasmosis to carry out for monitoring the possible dissemination of toxoplasmosis during hemodialysis.

## Introduction

*Toxoplasma gondii* is an obligate and intracellular parasite, extensively spread worldwide and capable to infect large variety of warm-blooded hosts.

Human *T*. *gondii* infection can results from consuming of food, soil and water harboring oocysts or infected raw or undercooked meat.

When this parasite infects humans, it can occasionally lead to abortion in expectant mothers and produce severe clinical symptoms in those with compromised immune systems. However, it is typically asymptomatic [1–3]. Systematic review and meta-analysis reported that the overall seroprevalence rate of toxoplasmosis among the general population in Iran was 39.3% (95% CI = 33.0–45.7%) [4]. Several studies have shown that individual with hemodialysis have a weak immune system and these patients are vulnerable to numerous opportunistic infections such as *T. gondii* [5, 6].

In immunocompromised patients, severe neurological symptoms are commonly showed consequences of reactivation of latent *Toxoplasma* infection [7, 8].

The majority of human and animal isolates fall into one of three categories: I, II, or III which in some regions of the world such as Europe and North America, the 4th lineage has also joined them [9]. While there is less than 1% genetic separation between these three dominant groups, the variance in mouse phenotypes is highly noticeable. When mice are infected with type II lineage of *T. gondii*, they survive and the tachyzoites are less invasive than when they are infected with type I, which is deadly to mice [10]. Type I and atypical species are only seen in severe toxoplasmic chorioretinitis and in immunocompromised patients, not in congenital toxoplasmosis or asymptomatic toxoplasmosis [11]. These three categories can all stimulate the immune system of the host and cause the generation of antibodies.

*Toxoplasma* genotypes have been determined using a variety of techniques, and many targets have been researched. Both injecting sensitive laboratory animals with the parasites and cultivating on living tissue are labor-intensive and insensitive ways for finding parasites in the blood. As a result, today, parasite detection in biological products uses the highly sensitive and specific PCR approach [12].

So far several studies were conducted to reveal the population structure of *T. gondii* by molecular methods. In comprehensive study based on phylogenetic analysis of more than 950 typed isolates through the word, 16 described haplogroups were recognized [13], and assorted into six main clades according to sequencing analyses manner [14].

Giving the important role of a genetic depiction of *T. gondii* isotypes in epidemiological and clinical scurvies, this study was aimed to evaluate the frequency and genotyping of *T. gondii* in hemodialysis patients (HP).

## Material and methods

### Patients and blood samples

**Fig. 1:**
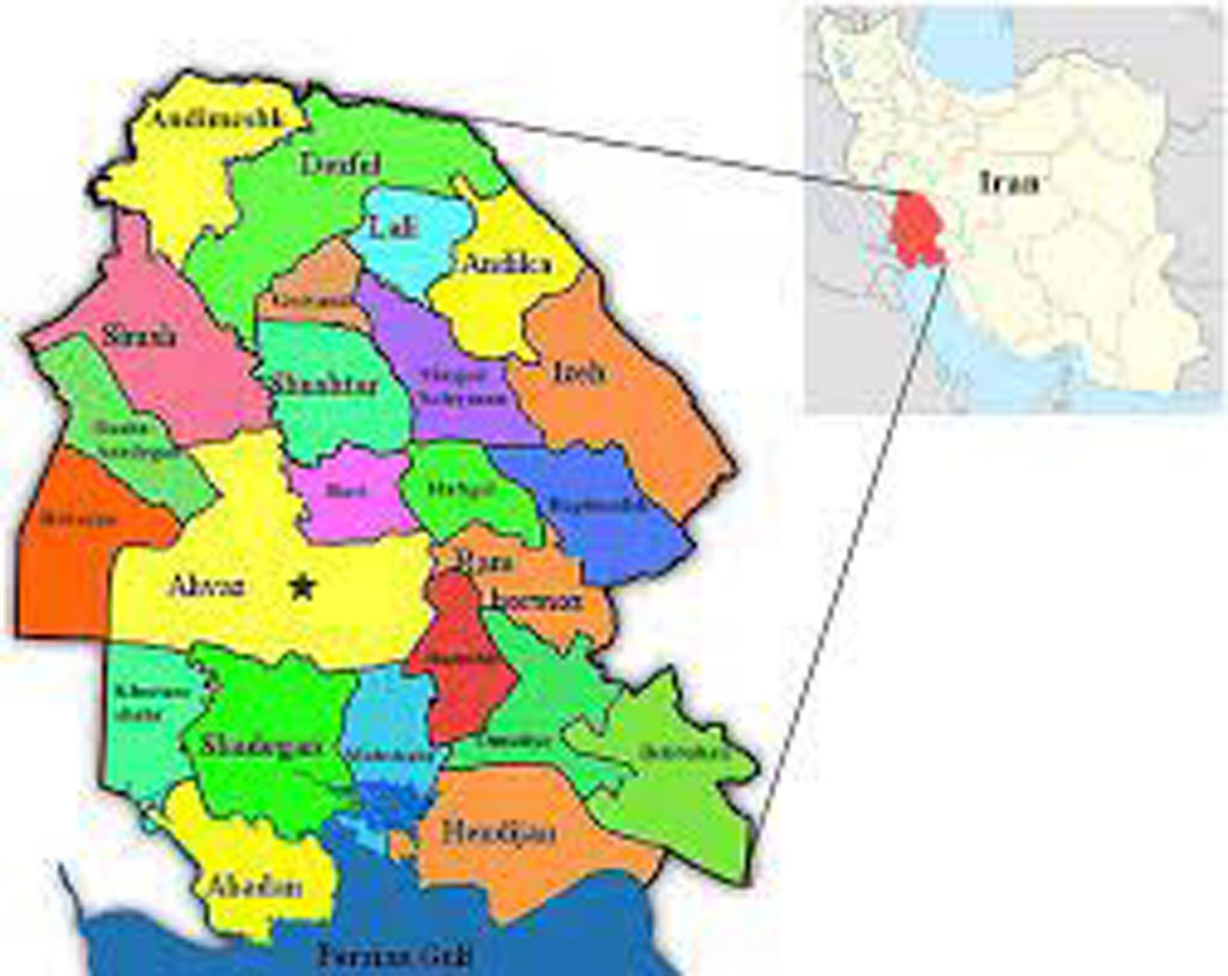
Map viewing the locality of study area

This descriptive cross-sectional survey was conducted in Khuzestan, southwest Iran, during the years 2018–2019 on patients with hemodialysis (Fig. 1). A total of 379 blood samples were taken from HP who were being treated at the Ahvaz Jundishapur University of Medical Sciences affiliated hospitals under the direction of urologists. The consent form was filled out by the patient or a family member for each patient. Patients with underlying diseases such as diabetes, cancer and other diseases affecting the immune system were excluded from the study. Patients whose hemodialysis started at least 6 months ago were included in the study. Heparinized blood was centrifuged at 5000 rpm for 5 min to harvest the buffy coat samples. Produced buffy coats were kept at − 20 °C until use.

### Ethics approval and consent to participate

The Ahvaz Jundishapur University of Medical Sciences Ethics Committee approved this survey (ethics code: IR.AJUMS.MEDICINE.REC. 1398.003). The consent form was completed and signed by all the participants or their relatives.

### DNA extraction

AccuPrep® was used to extract the DNA from Buffy coat. A PCR test was run on the DNA using a genomic DNA extraction kit (South Korea). PCR results were read by Bio-Rad GEL DOC after electrophoresis on a 2% agarose (Sigma; Australia) gel (Minneapolis, Minnesota, United States).

### Nested PCR

Nested PCR was carried out using two distinct primer pairs for the B1 gene in order to detect B1 *T. gondii* DNA in buffy coats. A pair of external primers, B1-external-forward-5’-TCAAGCAGCGTATTGTCGAG-3’ and B1-external-reverse-5’-CCGCAGCGACTTCTATCTCT-3’ were used for the first round and two internal primers, B1-internal-forward-5’-GAACTGCATCCGTTCATGAG-3’ and B1-internal-Reverse-5’-TCTTTAAAGCGTTCGTGG TC-3’ were used for the second round to amplify 194 bp fragment [15].For PCR, a combination of 10 μl of Master Mix buffer (2X, Ampliqon, South Korea), 2 μl (mM) of each primer, 3 μl of distilled water, and 3 μL of template DNA were used. To carry out nested-PCR, 2 μL of the PCR product as a template diluted in distilled water at a ratio of 1:10, and was performed according to the previous step. For positive and negative control of the reactions, the RH strain of *T. gondii* and the DNA-free reaction was used, respectively. Amplification steps were performed using thermal cycler (Eppendorf AG 22331, Hamburg, Germany) according to the following pattern: 5 min at 94 °C for initiation of denaturation, 30 cycles for 25 s at 94 °C, annealing at 53 °C for 20 s at both PCR and nested PCR, in 72 °C for 20 s extension and 5 min at 72 °C for final extension. An amount of 10 μL of each sample of the nested-PCR product was electrophoresed on a 2% (w/v) agarose gel and visualized by staining with DNA Safe Stain and then observed under ultraviolet light [16].

### DNA sequencing and phylogenetic analysis

Secondary PCR products from nine positive isolates were randomly chosen, together with a positive control, and sequenced in Spain (NIMGenetics) to verify the findings and genetic analyses. ChromasPro software was used to evaluate the chromatograms (version 2.1.4). Multiple sequence alignments were created using the Molecular Evolutionary Genetics Analysis (BioEdit) software (version 7.2), and the sequences were then checked against the GenBank database using a BLAST search for nucleotide sequence homology on the network server of the National Center for Biotechnology Information (NCBI). The B1 rDNA gene sequences from our products and those found in GenBank were used to construct a phylogenetic tree of *T. gondii*.

### Data analysis

Epi Info 7 was used to compare the percentages of infection prevalence. Using the Chi-square test, the findings were statistically analyzed.

*p* values of 0.05 or above were regarded as statistically significant.

## Results

A total of 379 patients receiving hemodialysis were enrolled in the trial. The individual's ages, which ranged from 18 to 70 years old, were 34.6(± 10.41) years on average.

**Fig. 2:**
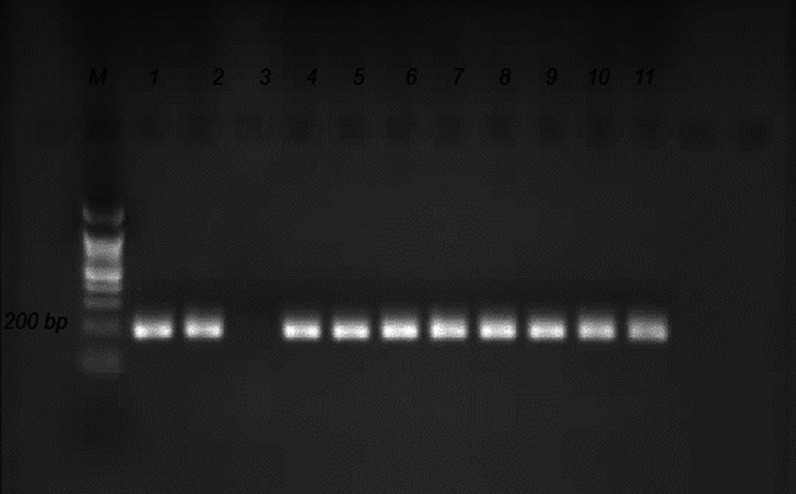
Nested PCR B1 electrophoresis on a 2% agarose gel, lanes M (a Ladder for 100 bp), lane 1 T*. gondii* RH strain positive control, Lane 2 and Lanes 4–11 are clinical isolates, and lane 3 the negative control (distilled water)

After the second cycle of Nested-PCR, the clinical samples produced the anticipated band of roughly 200 bp, as shown in Fig. 2, with no band in the negative control. Using specified primers, the B1 gene was amplified by PCR, yielding 112 T*. gondii* DNA fragments with an average length of 200 bp.

The B1 gene PCR-amplified results were then sequenced. These sequences were registered in DNA Databank of Japan (DDBJ) under accession numbers of LC789536- LC789545). Based on genetic changes in the B1 gene sequence, the comparison and molecular phylogeny of the nine hemodialysis isolates, as well as their identities and potential connections to other representative *T. gondii* from different countries, were assessed.

**Fig. 3:**
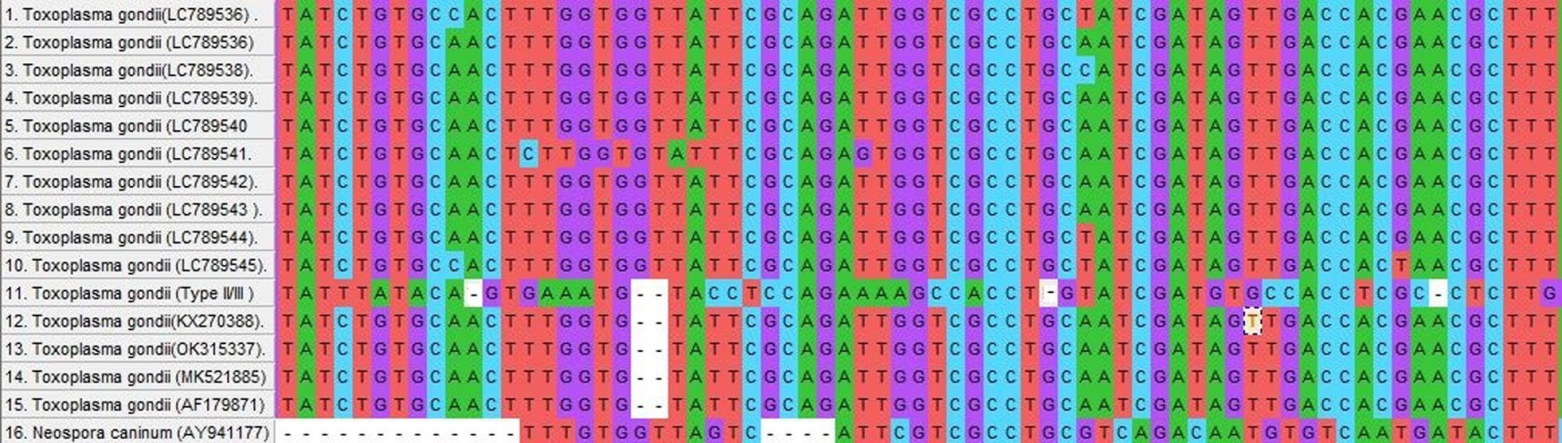
Nucleotide sequences of the *T. gondii* B1 gene from nine isolates (LC789536–LC789545) have been aligned numerous times using reference sequences (MK521885, KX270388 and OK315337). LC789538 related to tachyzoites of *T. gondii* RH strain is kept in the laboratory

It was established by alignment that *T. gondii* belonged to type I isotype after the retrieved nucleotide sequences shown 100% homology in contrast to the other published sequences in GenBank. The similarities and differences between these sequences and other GenBank-recorded sequences were revealed by multiple sequence alignment (Fig. 3).

**Fig. 4:**
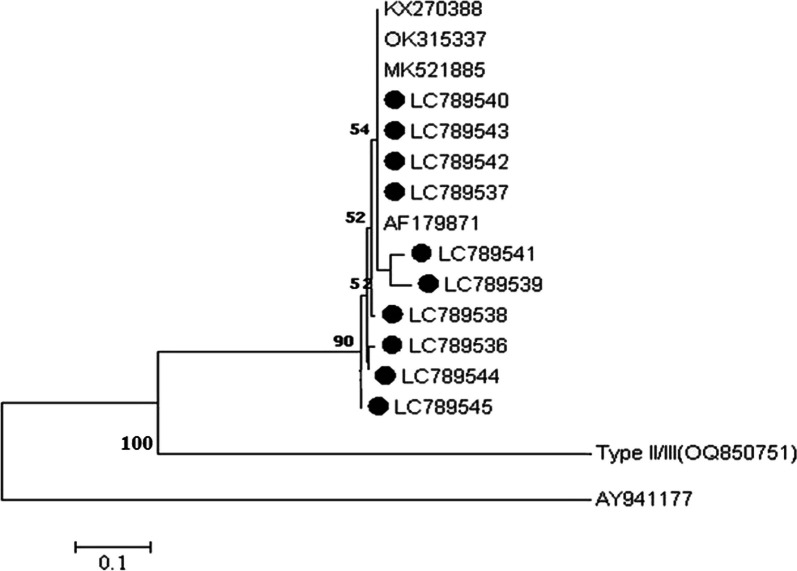
The phylogenetic analysis was conducted on the B1 gene of *Toxoplasma gondii* in hemodialysis patients from Khuzestan, southwestern Iran. The analysis was based on the maximum likelihood method using the Tamura-Nei model. The black circles represent the B1 sequences generated in this study. To compare the results, previously published sequences of humans and animals’ origin were retrieved from GenBank and included in the analysis. *Neospora caninum *(AY941177) was used as the outgroup taxa. Evolutionary analyses were conducted in MEGA7. LC789538 related to tachyzoites of *T. gondii* RH strain is kept in the laboratory

The *T. gondii* isolates under investigation are based on a branch in phylogenetic analysis that characterizes type I and is similar to sequences seen in other countries (Fig. 4).

## Discussion

In recent years, the number of people with kidney failure who need hemodialysis has increased. Iran currently has over 13,000 dialysis patients who receive treatment three times a week to maintain their lives, and another 17% are added each year. Additionally, over 14,000 patients received kidney transplants [17]. Due to the weakness of the immune system, especially cellular immunity, these people suffer from infections caused by opportunistic agents and need special care and attention. *T. gondii* as an opportunistic protozoan can threaten the health of these people. The parasite has three main clonal lineages (types I, II and III), which are different in terms of pathogenicity, type I much more virulent for mice than type II and III strains. There is a lack of knowledge about the kind type of *T. gondii* infection among patients undergoing hemodialysis in Southwest of Iran. Thus, in the current study, we evaluated the frequency of *T. gondii* DNA in HD and confirmed the genotype of the parasite.

In the current investigation, 112 (29.55%) samples from the HP group contained circulating *T. gondii* parasite DNA for gene B1. The values of the study findings are more than certain stated numbers and lower than others in comparison. In Arab-Mazar et al. investigation in Tehran the capital of Iran,* Toxoplasma* DNA was discovered in two of the 18 blood samples from IgM-positive HP in, but not in any of the IgM-positive control participants [16].

In study conducted in Zahedan City, southeastern Iran, in HP with chronic renal disease, *T. gondii* DNA was detected 29.4%, in the case group while the comparable values in the control group was 2.52% (*p* < 0.05) [18]. Mirahmadi et al. found that 49/106 (46.23%) and 3/106 (2.84%) of HP and healthy persons, respectively, were positive for the RE gen of *T. gondii* by real-time PCR (*p* < 0.05) [19]. In a prior investigation, we demonstrated that PCR did not produce positive results in healthy control group but did in 1.4% (4/280) of HP patients [20].

Five samples (6%) were found to have *Toxoplasma* DNA positive by PCR in the study published by Rezavand et al. [21]. The phylogenetic analysis revealed that all *isolates* were closely related to Type I of *T*. *gondii*. Type I strains are linked to congenital toxoplasmosis, cerebral toxoplasmosis, and acquired ocular toxoplasmosis in immunocompromised patients, according to surveys on human toxoplasmosis in Europe and the USA [22]. Fuentes et al. reported strains of *T. gondii* type II were the most prevalent in immunocompromised (HIV +) patients, with 52% of cases, while strains of type I were present in 75% of the congenital infection cases [23].

Sabzevari et al. indicated all cases of 16 CSF samples were found positive by the nested-PCR method. After genotyping CSF samples using RFLP assay, *T. gondii* type II was found in 15 samples and one sample was a mix of both types II and III [24].

*T. gondii* oocysts, which can infect people and animals through the environment, including contaminated foods, water, or soil, can have an impact on the prevalence of *T. gondii* infection in a given location. Studies carried out in Iran revealed various genotypes in various racial and ethnic groups. In their molecular detection and genotyping of *T. gondii* in Chicken, Beef, and Lamb meat consumed in Northwestern Iran study, Mahami-Oskouei et al. revealed that 17.33% of the samples were positive for *T. gondii*, including 8% from Chicken, 16% from Cattle, and 28% from Sheep. Additionally, they stated that all of the samples were genotype I. [25]. In a study on environmental soil samples in Mazandaran Province, North of Iran, Haghparast-Kenari et al. identified 23 samples as 91.3% having type I genotype and 8.7% having type II genotype [26]. While genotype I was reported as the most common genotype in the prior study, which genotyped *T. gondii* in HP in various locations throughout the world using the B1 and Nested PCR method, some researchers disagree with this overused claim. They identified no type I strain in these patient groups, i.e., a RH-like strain, according to their studies. Contrarily, type II strains predominate in immunocompromised persons regardless of the underlying cause of immunosuppression, the site of infection, or the result [27]. *Toxoplasma* strain genotype, however, is difficult to predict based on the suspected geographic source of infection: immunocompromised individuals typically reactivate a type II strain if infected in Europe and an atypical strain if acquired in sub-Saharan African countries [27].

The strengths of this study were, subjects under study and direct detection of parasite-specific DNA in all blood samples by Nested PCR method, regardless of the negative or positive serological result. In addition, the limitations of the study was failure to follow*-*up the patients. In conclusion, the findings indicated that the frequency of toxoplasmosis in HP was relatively high. In addition, current study indicated that the genotype I was the prevailing genotype in the patients. Giving HP are immunocompromised and *T. gondii* poses a serious threat to the patients, we suggest that periodic monitoring for *T. gondii* infection and genotyping should be combined into the routine clinical care of the patients.

## Data Availability

All data supporting the findings of this study are available within the paper. No additional documents is created other than which is listed in manuscript, table and figures.
